# NRF2 Orchestrates the Metabolic Shift during Induced Pluripotent Stem Cell Reprogramming

**DOI:** 10.1016/j.celrep.2016.02.003

**Published:** 2016-02-18

**Authors:** Kate E. Hawkins, Shona Joy, Juliette M.K.M. Delhove, Vassilios N. Kotiadis, Emilio Fernandez, Lorna M. Fitzpatrick, James R. Whiteford, Peter J. King, Juan P. Bolanos, Michael R. Duchen, Simon N. Waddington, Tristan R. McKay

**Affiliations:** 1 Stem Cell Group, Cardiovascular and Cell Sciences Research Institute, St. George’s University of London, Cranmer Terrace, London SW17 0RE, UK; 2 Department of Cell and Developmental Biology, University College London, Gower Street, London WC1E 6BT, UK; 3 Wits/SAMRC Antiviral Gene Therapy Research Unit, Faculty of Health Sciences, University of the Witwatersrand, Johannesburg 2000, South Africa; 4 Gene Transfer Technology Group, Institute for Women’s Health, University College London, 86-96 Chenies Mews, London WC1E 6HX, UK; 5 Institute of Functional Biology and Genomics, University of Salamanca-CSIC, 37007 Salamanca, Spain; 6 Institute of Biomedical Research of Salamanca, University Hospital of Salamanca, 37007 Salamanca, Spain; 7 William Harvey Research Institute, Charterhouse Square, Queen Mary University of London, London EC1M 6BQ, UK; 8 School of Healthcare Science, John Dalton Building, Manchester Metropolitan University, Chester Street, Manchester M1 5GD, UK

## Abstract

The potential of induced pluripotent stem cells (iPSCs) in disease modeling and regenerative medicine is vast, but current methodologies remain inefficient. Understanding the cellular mechanisms underlying iPSC reprogramming, such as the metabolic shift from oxidative to glycolytic energy production, is key to improving its efficiency. We have developed a lentiviral reporter system to assay longitudinal changes in cell signaling and transcription factor activity in living cells throughout iPSC reprogramming of human dermal fibroblasts. We reveal early NF-κB, AP-1, and NRF2 transcription factor activation prior to a temporal peak in hypoxia inducible factor α (HIFα) activity. Mechanistically, we show that an early burst in oxidative phosphorylation and elevated reactive oxygen species generation mediates increased NRF2 activity, which in turn initiates the HIFα-mediated glycolytic shift and may modulate glucose redistribution to the pentose phosphate pathway. Critically, inhibition of NRF2 by KEAP1 overexpression compromises metabolic reprogramming and results in reduced efficiency of iPSC colony formation.

## Highlights

• Cells increase proliferation, OXPHOS, and ROS production early in reprogramming
• The antioxidant response is therefore active at this stage, prior to HIFα activation
• NRF2 promotes HIFα activation, the metabolic switch, and colony formation
• NRF2 activation is concomitant with glucose redistribution to the PPP

## Graphical Abstract

**Figure:**
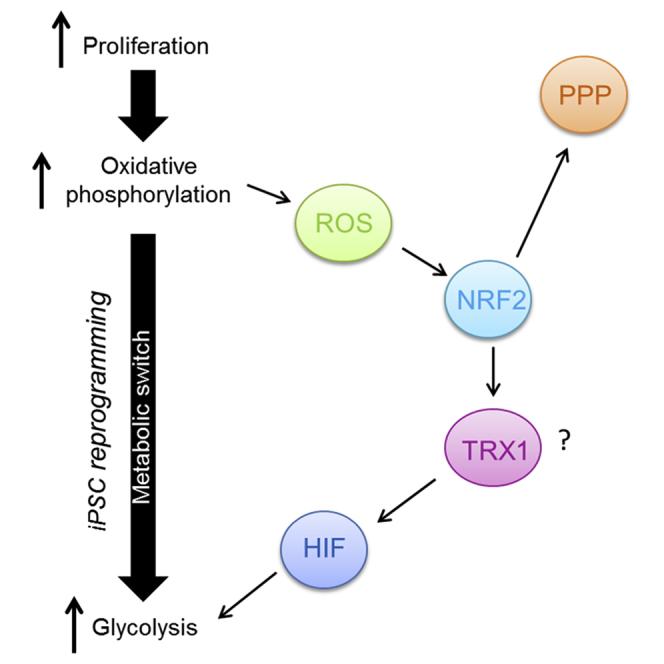

## Teaser

Hawkins et al. examine the metabolic shift during iPSC reprogramming. They propose that increased proliferation of cells driven by transgene expression can lead to increased oxidative phosphorylation resulting in ROS production. Elevated ROS activates NRF2, promoting HIFα activation and the switch to glycolysis.

## Introduction

The ability to genetically reprogram a somatic cell to an induced pluripotent stem cell (iPSC) represented a paradigm shift in stem cell research upon its first description (Takahashi and Yamanaka, 2006) and provides great promise for regenerative medicine, but the process remains inefficient. It has been proposed that iPSC reprogramming is a stochastic process (Hanna et al., 2009), but there is emerging evidence that it is deterministic with initiation, stabilization, and maturation stages (Golipour et al., 2012, Samavarchi-Tehrani et al., 2010) involving the coordinated temporal activation and repression of cell signaling pathways (Park et al., 2014, Polo et al., 2012). Reprogramming cells undergo profound changes in morphology, function, and metabolic activity with somatic cells that predominantly rely on mitochondrial respiration to produce ATP, switching to glycolysis (Folmes et al., 2011, Panopoulos et al., 2012, Prigione et al., 2010, Varum et al., 2011). The opposite transition has also been shown to occur during differentiation of human embryonic stem cells (hESCs; Cho et al., 2006) and involves mitochondrial biogenesis. However, upon reprogramming, human dermal fibroblast (hDF) mitochondria acquire immature morphological features typical of those observed in hESCs (Lonergan et al., 2006, Prigione et al., 2010), although their relative density as a ratio to cytoplasmic volume remains broadly the same (Zhang et al., 2011a).

Many stem cells, including hESCs, maintain quiescence and potency in a physiologically hypoxic niche in vivo (Danet et al., 2003, Ezashi et al., 2005, Morrison et al., 2000, Studer et al., 2000). Furthermore, iPSC reprogramming (Shimada et al., 2012, Yoshida et al., 2009) and the maintenance of hESC lines (Chen et al., 2010) are enhanced under hypoxic conditions. Hypoxia inducible factor-α (HIFα) transcription factor activity stimulates glycolytic gene expression in adult stem cells (Palomäki et al., 2013) and cancer stem cells (Finley et al., 2011) and occurs during iPSC reprogramming (Prigione et al., 2014), with two recent studies indicating that HIFα activation is integral to the upregulation of glycolysis in the initiation stages of iPSC reprogramming independent of oxygen tension (Prigione et al., 2014, Mathieu et al., 2014). Specifically, Mathieu et al. (2014) show that ectopic expression of the isoform HIF1α throughout iPSC reprogramming promotes colony formation, whereas HIF2α overexpression enhances the early stages but is inhibitory in the later phases.

A major limitation in the study of transcription factor activity driving metabolic reprogramming during iPSC generation, stem cell differentiation, or tumor initiation is the ability to quantitate activity in living cells. To date, only end-point or semiquantitative fluorescent protein analyses have been employed in mechanistic investigations of iPSC reprogramming (Hansson et al., 2012, Samavarchi-Tehrani et al., 2010). Here we utilize a dual-reporter system where secreted NanoLuc luciferase (NLuc) and eGFP are expressed under the conditional control of a transcription factor activated reporter (TFAR) and normalized for cell proliferation against a second constitutively active secreted *Vargula* luciferase (VLuc). Using this method, we are able to monitor transcription factor activity in live cell cultures throughout iPSC reprogramming.

From an initial screen of eight candidate transcription factors or cell signaling pathways known to play a role in iPSC reprogramming, we found a reproducible temporal wave of nuclear factor kappa B (NF-κB), activator protein 1 (AP-1), and nuclear factor (erythroid-derived 2)-like 2 (NRF2) activity prior to a distinct HIFα peak, which correlated with the metabolic shift toward glycolysis. NRF2, which is upregulated within 2 days of iPSC reprogramming, is a master regulator of the stress response, particularly to reactive oxygen species (ROS), and its activation is complex and multifactorial. Under conditions of homeostasis, NRF2 forms proteasomal degradation complexes with two E3 ubiquitin ligase adaptors: Kelch-like ECH-associated protein 1 (KEAP1) and β-TrCP. Whereas p62/SQSTM1 competes with NRF2 for binding to KEAP1, thus activating NRF2 signaling (Hayashi et al., 2015, Ichimura et al., 2013), glycogen synthase kinase-3β (GSK-3β) increases the binding of β-TrCP to NRF2, thus resulting in ubiquitination and proteasomal degradation of NRF2 (Chowdhry et al., 2013). ROS exposure causes cysteine modifications in KEAP1, allowing newly translated NRF2 to evade ubiquitination and thus mediate activation of genes containing antioxidant response elements in their promoters (Baird et al., 2013, McMahon et al., 2006).

We show a longitudinal profile of NRF2 activity during iPSC reprogramming peaking at day 8 prior to initiation of a HIFα-mediated glycolytic shift and thereafter decreasing to basal levels. In contrast to the existing dogma, we show that in the early stages of reprogramming, highly proliferative cells actually increase mitochondrial respiration as well as channeling glucose to the pentose phosphate pathway (PPP) to manage increased nucleotide synthesis demands. The peaks in cell proliferation, oxidative phosphorylation (OXPHOS), and PPP all correlate with maximal NRF2 activity. Glycolysis increases in response to a transient HIFα peak, which is in itself dependent on NRF2 activity. Our data indicate that NRF2 activity is primarily affected through increased ROS production in this context and can be reversed by KEAP1 overexpression, which inhibits metabolic reprogramming and results in drastically reduced iPSC colony formation. We conclude that NRF2 acts at a critical nexus between coordinating the distribution of glucose between catabolism and anabolism while managing the stress response and initiating the metabolic switch during the initiation stages of iPSC reprogramming.

## Results

### TFAR Lentiviral Transduction for Real-Time Quantification of Transcription Factor Activity during iPSC Reprogramming

**Figure 1:**
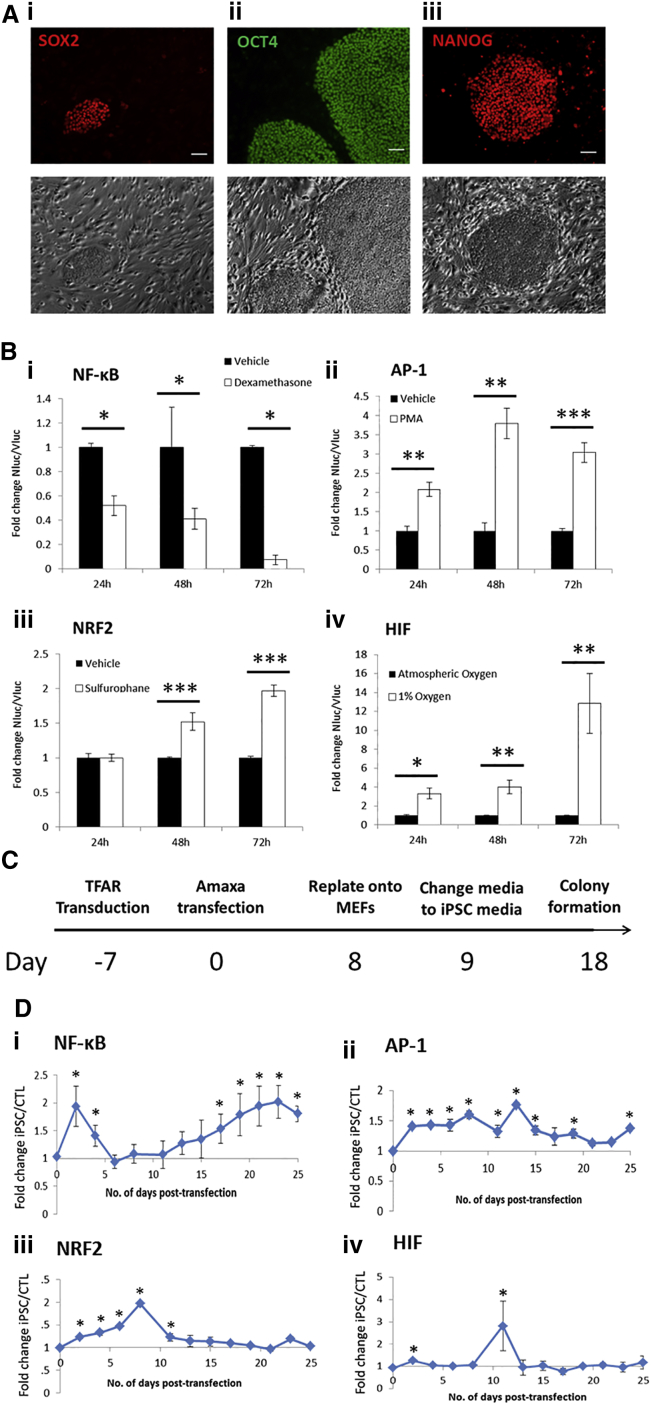
Validation of Methodology (A) Immunofluorescent cell staining for pluripotency marker expression. (B) Validation of TFARs in hDFs. (C) Schematic of the iPSC reprogramming protocol. (D) Graphs to show fold change of normalized TFAR activity for iPSCs compared with control cells, n = 9. Scale bars represent 100 μm. MEF, mouse embryonic fibroblast; LTR, long terminal repeat; PMA, phorbol myristate acetate. ^∗^p < 0.05, ^∗∗^p < 0.01, ^∗∗∗^p < 0.005. Error bars represent SEM for three biological replicates. See also Figures S1 and S3.

In this study, we chose to use the latest iteration of the Yamanaka iPSC reprogramming methodology employing episomally maintained plasmids (Yu et al., 2009). iPSCs generated using this protocol were shown to exhibit pluripotent morphology (Figure S1Ai), pluripotency-associated gene expression (Figure S1Aii), and protein expression (Figure 1A). iPSCs from this protocol also formed embryoid bodies in vitro (Figures S1Bi–iii) and teratomas in NOD/SCID mice (Figures S1Ci–iii) containing tissues representative of all three germ layers.

We designed and produced seven TFAR lentiviral vectors containing synthetic promoters activated by cell signaling pathways previously implicated in iPSC reprogramming (Figure S1Di; sequences and validation can be found in Buckley et al., 2015). For clarity, the “AP-1” synthetic promoter consists of eight repeats of the sequence TGAGTCAG; thus, the TFAR can be activated by either c-Fos/c-Jun heterodimers or c-Jun/c-Jun homodimers (Park et al., 2003). We also included a reporter vector with a truncated version of the ICAM1 promoter because O’Malley et al. (2013) have previously reported a critical temporal role for ICAM1 expression in the early stages of mouse iPSC reprogramming. The lentiviral expression cassettes express secreted NLuc and are based on our previously described vectors (Buckley et al., 2015). In order to control for cell proliferation when using genome-integrating vectors, we developed a second constitutively active lentiviral vector expressing the secreted VLuc (Figure S1Dii). NLuc and VLuc have unique non-overlapping substrates whose activity is independent of ATP. Specificity of our TFAR was confirmed in transduced hDFs (<10 multiplicity of infection [MOI]) exposed to relevant pathway agonists and antagonists (Table S1). Expression of eGFP fluorescence and NLuc/VLuc luciferase activity was assayed in conditioned medium over 72 hr (Figures 1Bi–iv and S2i–viii). All TFARs demonstrated modulation of eGFP expression and significant changes in NLuc/VLuc ratio within this timeframe.

TFAR activity was assayed throughout iPSC reprogramming using the protocol shown in Figure 1C. NLuc/VLuc activity was quantified in conditioned medium and expressed as a fold change over NLuc/VLuc activity in control cultures transfected with equivalent molar quantities of empty episomal plasmid. Quality control was determined by the required emergence of more than ten colonies per 1 × 10^5^ cells after 25-days post-transfection. FOXO and ICAM1 reporters showed no significant changes in activity, but NFAT and NOTCH both showed persistent repression during iPSC reprogramming compared with controls from day 11 (Figures S3i–iv). Most intriguingly, we observed early and significant increases in NF-κB, AP-1, NRF2, and HIFα TFAR activity (Figure 1Di–v), transcription factors previously associated with the stress/antioxidant response (Gonchar and Mankovska, 2010). Increased activity of these TFARs was validated by the concomitant increased expression of established target genes at day 2 (Figure S3v) and the observation of nuclear localization of c-Fos protein at day 4 in reprogramming cells (Figure S3vi).

**Figure 2:**
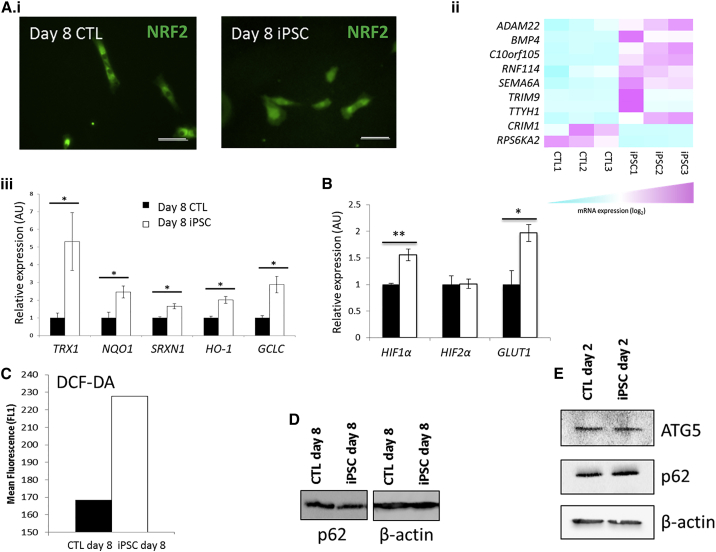
Confirmation of TFAR Activation Data (A) (i) Immunofluorescent cell staining to show NRF2 is localized in the nucleus of pre-iPSCs but largely excluded from the nucleus of control cells at day 8 of reprogramming. (ii) Heatmap to show significantly altered NRF2 target gene expression in iPSCs and control cells at day 8 of reprogramming by RNA-seq. (iii) qPCR to show upregulation of NRF2 target genes at day 8 of iPSC reprogramming compared to control cells. (B) qPCR to show upregulation of *HIF1α* and its target *GLUT1* at day 11 of iPSC reprogramming. (C) Flow cytometry of DCF-DA to show increased ROS in pre-iPSCs at day 8 of reprogramming compared with control cells. (D) Western blot analysis of p62 protein expression at day 8 of iPSC reprogramming. (E) Western blot analysis of p62 and ATG5 transcript expression at day 2 of reprogramming. n = 3 for all. Scale bars represent 100 μm. ^∗^p < 0.05, ^∗∗^p < 0.01. Error bars represent SEM for three biological replicates. ADAM22, A disintegrin and metalloprotease domain 22; BMP4, bone morphogenetic protein 4; c10orf105, chromosome 10 open reading frame 105; RNF114, ring finger protein 114; SEMA6A, semaphorin-6A; TRIM9, tripartite motif containing 9; TTYH,: Tweety family member 1; CRIM1, cysteine-rich transmembrane BMP regulator 1; RPS6KA2, ribosomal protein S6 kinase; TRX1, thioredoxin 1; NQO1, NAD(P)H dehydrogenase quinone 1; SRXN1, sulfiredoxin 1; HO-1, heme oxygenase 1; GCLC, glutamate-cysteine ligase catalytic subunit. See also Figures S2 and S3.

In this study, we focused on the peak in NRF2 activity at day 8 of iPSC reprogramming since NRF2 is the master regulator of the antioxidant response. At this time point, NRF2 is localized in both the cytoplasm and nucleus in reprogramming cells but is largely excluded from the nucleus in control cells (Figure 2Ai). An RNA-seq comparison of reprogramming and control cells at day 8 also showed NRF2 target gene transcripts to be significantly upregulated in reprogramming cells (Figure 2Aii). This was also confirmed at the molecular level since the NRF2 target genes thioredoxin 1 (*TRX1*), NAD(P)H dehydrogenase quinone 1 (*NQO1*), sulfiredoxin 1 (*SRXN1*), heme oxygenase 1 (*HO-1*), and glutamate-cysteine ligase catalytic (*GCLC*) subunit were significantly upregulated in reprogramming cells compared with control cells at day 8 (Figure 2Aiii). Consistent with our TFAR data during iPSC reprogramming, *HIF1α* and its glycolytic target *GLUT1* were significantly upregulated at day 11 compared with controls. Interestingly, *HIF2α* transcript expression was not significantly altered in reprogramming cells compared with control cells at day 11 of reprogramming (Figure 2B). These data are consistent with the observations of Mathieu et al. (2014).

We hypothesized that the early increase in NRF2 activity was in response to elevated ROS generated from high levels of mitochondrial activity in reprograming cells, so we analyzed ROS levels using flow cytometry for 2′,7′-dichlorofluorescin diacetate (DCF-DA) at day 8 of iPSC reprogramming. Levels of ROS were indeed higher in reprogramming cells compared with control cells (Figure 2C). In addition to ROS, NRF2 can be activated by the autophagy-associated p62 protein. There was no quantifiable difference in p62 protein in lysates from iPSC reprogramming cells either at day 2 or day 8 and no quantifiable change in the autophagy-associated ATG5 protein at day 2 (Figures 2D–2E). Additionally, we found no difference in the levels of transcript expression of the NRF2 repressor protein KEAP1 at this time point (Figure S3vii), thus suggesting that KEAP1 regulation is post-translational. This is consistent with our hypothesis that modification of cysteine residues of KEAP1 by ROS causes NRF2 activation at day 8 of iPSC reprogramming.

### Reprogramming Cells Temporarily Increase OXPHOS and PPP Activity

**Figure 3:**
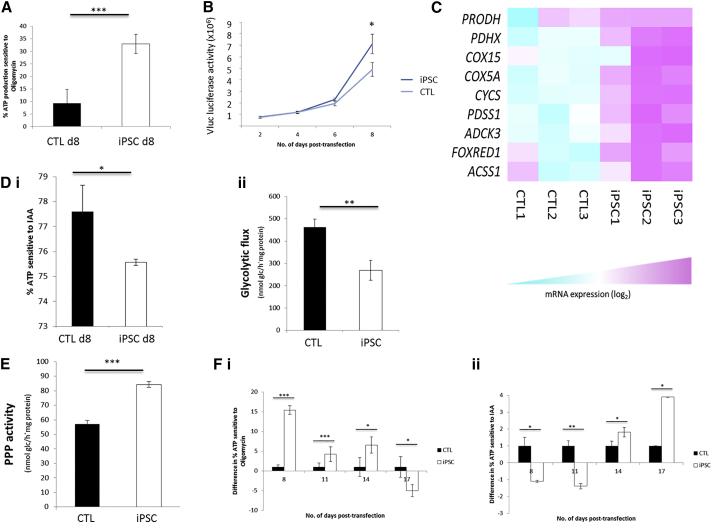
Reprogramming Cells Experience Transient Increases in OXPHOS and PPP Flux and Decreases in Glycolysis (A) ATP assay to show increased levels of ATP production by OXPHOS in iPSCs compared with control cells at day 8 of reprogramming. (B) VLuc luciferase activity over time in pre-iPSCs and control cells. (C) RNA-seq to show levels of expression of significantly altered OXPHOS-related genes in control cells versus iPSCs. (D) (i) ATP assay to show decreased levels of ATP production by glycolysis at day 8 of reprogramming. (ii) Decreased glycolytic flux in iPSCs compared with control cells. (E) Increased PPP activity in reprogramming cells. (F) ATP assays to show levels of OXPHOS (i) and (ii) glycolysis throughout reprogramming. n = 3 for all. Error bars represent SEM for three biological replicates. ^∗^p < 0.05 ^∗∗^p < 0.005, ^∗∗∗^p < 0.001. PRODH, proline dehydrogenase (oxidase) 1; PDHX, pyruvate dehydrogenase complex component X; GRPEL1, GrpE-like 1; COX15, cytochrome c oxidase assembly homolog 15; COX5A, cytochrome c oxidase subunit Va; CYCS, cytochrome c; AK4, adenylate kinase 4; MARS2, methionyl-tRNA synthetase 2; CLPB, ClpB caseinolytic peptidase B; PDSS1, prenyl (decaprenyl) diphosphatase synthase, subunit 1; ADCK3, aarF domain-containing kinase 3; FOXRED1, FAD-dependent oxidoreductase domain containing 1; ACSS1, acyl CoA synthetase short-chain family member 1; CAT, catalase; BCL2L13, BCL2-like 13; SLC22A4, solute carrier family 22, member 4; COX7A1, cytochrome c oxidase subunit VIIA, polypeptide 1 (muscle). See also Figure S3.

If the observed elevated ROS levels were due to increased mitochondrial respiration during the early stages of iPSC reprogramming, we would expect OXPHOS-mediated ATP production to be increased. We used a luciferase assay to determine levels of ATP produced when ATP synthase (Complex V), and therefore ATP production by OXPHOS, was inhibited using oligomycin A. We observed significantly higher levels of OXPHOS in reprogramming cells compared with control cells at day 8 of reprogramming (Figure 3A). This was also demonstrated by the increased rates of routine and maximal oxygen consumption, after injection of the uncoupling agent carbonyl cyanide 4-(trifluoromethoxy) phenylhydrazone (FCCP), observed in pre-iPSCs compared with controls at day 8 of iPSC reprogramming (Figure S3viii). This increase in mitochondrial OXPHOS activity and capacity early in iPSC reprogramming correlated with a significant increase in cell proliferation (Figure 3B) and is consistent with associated increased metabolic demands.

Interestingly, this increase in OXPHOS at day 8 of iPSC reprogramming is supported by our RNA-seq data within which there is a substantial enrichment of transcripts encoding OXPHOS-related proteins at this time point (Figure 3C). Intriguingly, we also observed decreases in glycolysis by both analysis of ATP production when glycolysis is blocked by idoacetate (IAA; Figure 3Di) and assessment of the rate of ^3^H_2_O production from 3-^3^H-glucose (Figure 3Dii) after day 8 of iPSC reprogramming. This would be consistent with glucose being shuttled away from the glycolytic pathway and toward the PPP. PPP activity was quantified by assessment of the difference between ^14^CO_2_ production from [1-^14^C]-glucose (which decarboxylates through the 6-phosphogluconate dehydrogenase-catalyzed reaction) and that of [6-^14^C]-glucose (which decarboxylates through the tricarboxylic acid cycle), as previously described (Herrero-Mendez et al., 2009, Larrabee, 1990). PPP flux increased concomitantly with the decrease in glycolytic flux after day 8 in pre-iPSCs compared with control cells (Figure 3E). Consistent with a programmed metabolic shift, increases in glycolysis in iPSCs became significant at day 14, after the HIFα TFAR peak, and decreases in OXPHOS only become significant by day 17 (Figures 3Fi and ii).

### NRF2 Activates HIFα and Drives the Metabolic Switch toward Glycolytic Energy Production

**Figure 4:**
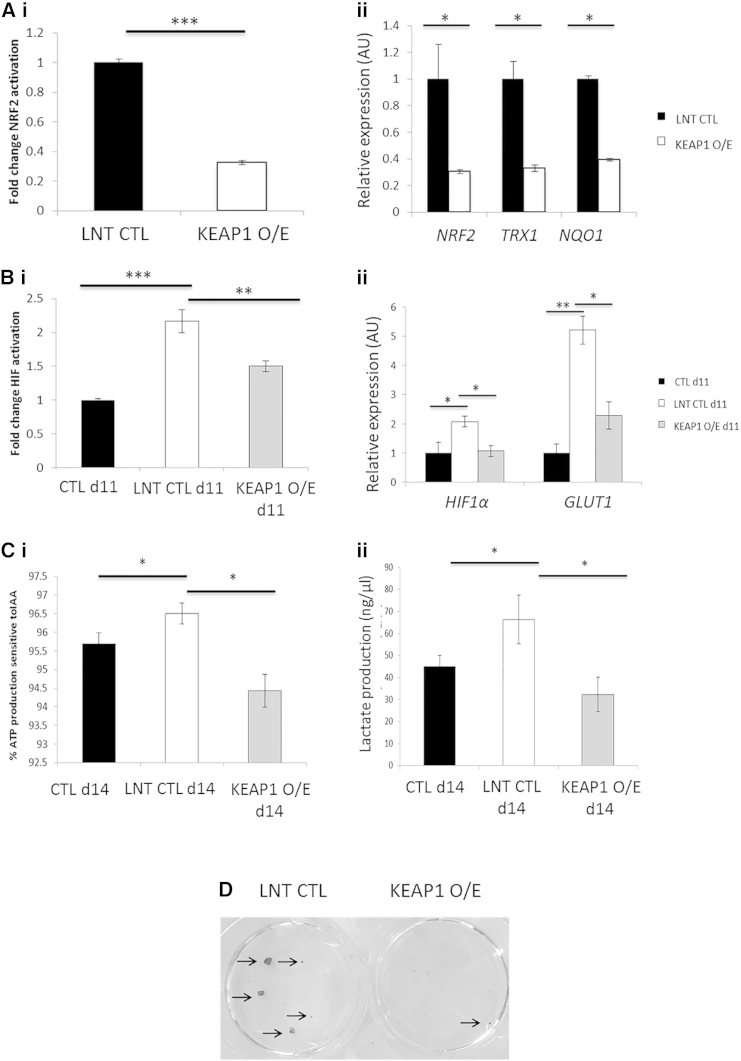
NRF2 Promotes Metabolic Reprogramming of iPSCs via HIFα (A) (i) Luciferase assay data to show decreased levels of NRF2 activity in hDFs transduced with a KEAP1 O/E lentivirus compared with a control lentivirus (LNT CTL). (ii) qPCR to show decreased expression of NRF2 targets in hDFs transduced with a KEAP1 O/E lentivirus. (B) (i) Luciferase assay data to show decreased levels of HIFα TFAR activity in KEAP1 O/E cells compared with LNT CTL cells at day 11 of iPSC reprogramming. (ii) qPCR to show decreased levels of HIFα target gene expression in KEAP1 O/E cells compared with LNT CTL cells at day 11 of iPSC reprogramming. (C) (i) ATP and (ii) lactate assays to show decreased levels of glycolysis in KEAP1 O/E cells compared to LNT CTL cells at day 14 of iPSC reprogramming. (D) Alkaline phosphatase staining of iPSC colonies. n = 3 for all. Error bars represent SEM for three biological replicates. ^∗^p < 0.05, ^∗∗^p < 0.005, ^∗∗∗^p < 0.001. O/E, overexpression. See also Figure S4.

Our data indicated a significant role for ROS-induced NRF2 in modulating the metabolic shift that occurs during iPSC reprogramming, so we generated a KEAP1-overexpressing lentiviral vector (KEAP1 O/E) to selectively inhibit NRF2 activity in transduced cells. The ability of KEAP1 O/E to decrease both NRF2 activity (Figure 4Ai) and target gene expression (Figure 4Aii) was confirmed in hDFs. We then subjected KEAP1 O/E and control empty vector transduced (LNT CTL) cells to iPSC reprogramming. KEAP1 O/E significantly inhibited HIFα TFAR activity at day 11 of reprogramming (Figure 4Bi) and reduced transcript levels of HIFα targets (Figure 4Bii). Furthermore, HIFα TFAR activity was significantly enhanced by activation of NRF2 either with deta NONOate, which induces mitochondrial ROS production (Jacobson et al., 2005; Figures S4i–iii) or an NRF2-overexpressing adenovirus (NRF2 O/E; Figures S4iv–vi). KEAP1 O/E also resulted in significantly lower levels of glycolysis, as assessed by luciferase ATP assay after inhibition with IAA (Figure 4Ci) and lactate production by day 14 of reprogramming (Figure 4Cii), whereas NRF2 activation either by deta NONOate (Figure S4vii) or by NRF2 O/E (Figure S4viii) resulted in early increases in the level of lactate production. Critically, KEAP1 O/E also resulted in a 5-fold decrease in iPSC colony formation (Figure 4D). Taken together, these data indicate that NRF2 promotes the metabolic shift from OXPHOS to glycolytic energy production during iPSC reprogramming via HIFα activation.

## Discussion

iPSC reprogramming is a fascinating biological phenomenon that we still know very little about. It remains debatable whether iPSC reprogramming is a stochastic series of events that concludes in colony formation or occurs in a deterministic stage-wise fashion. In this longitudinal study of transcription factor activity in hDF cell cultures during iPSC reprogramming, we assessed the activity of seven transcription factors and ICAM1 gene regulation. ICAM1 was included due to the observations of O’Malley et al. (2013) that mouse embryonic fibroblasts obtaining a CD44^−^/ICAM1^+^ phenotype during iPSC reprograming more efficiently transition to Nanog^+^ iPSC colonies. In contrast to this group, we did not observe modulation of the ICAM1 promoter during human iPSC reprogramming. This may be because O’Malley et al. (2013) quantified cell surface protein rather than transcriptional activation or may be due to species differences between mouse and human cells. Interestingly, we also did not detect any modulation of FOXO activity despite it being implicated in establishing the pluripotent state in hESCs (Zhang et al., 2011b). However, we did observe significant changes in six TFARs; four increased and two decreased their activity during iPSC reprogramming compared with controls. Levels of NFAT and NOTCH activity were lower during iPSC reprogramming compared with control sham reprogramming, which is consistent with previous reports demonstrating that inhibition of these pathways promotes pluripotency or self-renewal (Ichida et al., 2014, Zhu et al., 2014). Most strikingly, we observed a reproducible temporal wave of NF-κB, AP-1, NRF2, and HIFα activity. All four TFARs were significantly upregulated by day 2 with NF-κB and HIFα dropping to control levels by day 4, while AP-1 remained significantly elevated compared with controls throughout. NRF2 and HIFα activity peaked at days 8 and 11, respectively, prior to falling back to control levels.

All four transcription factors with increased activity during iPSC reprogramming are associated with oxidative stress responses (Reuter et al., 2010), so we reasoned that their increased activity was due to elevated ROS. As expected, ROS levels were higher in iPSC reprogramming compared with controls at day 8 when NRF2 activity was at its peak. Our data corroborate previous findings of high levels of ROS production in the early stages of reprogramming in mouse (Esteban et al., 2010) and human (Ji et al., 2014) cells. Crucially, we show that mitochondrial respiration is amplified during iPSC reprogramming up to day 14. This is in agreement with, but extends the observations of, Prigione et al. (2014), who showed that mitochondrial respiration increased over the first 3 days of reprogramming. Numerous studies have shown that c-MYC can induce ROS production (Esteban et al., 2010, Ji et al., 2014) and conversely that ROS can cause differentiation of hESCs (Ji et al., 2014). Furthermore, this has led to speculation that antioxidants may increase the efficiency of iPSC reprogramming by counteracting differentiation or preventing ROS-induced damage (Mah et al., 2011). For example, antioxidant supplementation with N-acetyl cysteine has been shown to promote cell survival and prevent double-strand DNA breaks (Ji et al., 2014), and supplementation with vitamin C has been shown to promote histone demethylation (Esteban et al., 2010). Our data showed that at the height of proliferative amplification reprogramming cells had higher OXPHOS relative to control cells but lower glycolysis. We rationalized that glucose was being redistributed in reprogramming cells and found that indeed the PPP was significantly increased in iPSC reprogramming compared with controls at the height of NRF2 activity. Others have shown that hESCs have an active PPP (Manganelli et al., 2012, Varum et al., 2011) and that mature iPSCs have increased PPP activity compared with their parental somatic cells (Varum et al., 2011). However, we specifically map PPP activation to the initiation stages of iPSC reprogramming and, crucially, demonstrate that this activation occurs prior to the metabolic switch from OXPHOS to glycolysis. Interestingly, and contradictory to its role in some adult stem cells (Tsai et al., 2013), NRF2 has been shown to increase the expression of components of the PPP in cancer cells to facilitate increased proliferation (Mitsuishi et al., 2012, Singh et al., 2013), suggesting that NRF2 may be playing a similar role in this context. This would also provide an additional mechanism by which NRF2 protects reprogramming cells from oxidative stress since the reducing agent NADPH is generated by PPP activity.

Our data imply that metabolic reprogramming occurs between day 8 and day 14 of iPSC reprogramming in this system. This is the period in which both NRF2 (day 8) and HIFα (day 11) activity peaks. NRF2 peak activity preceded that of HIFα, so we sought to investigate a functional link between the two transcription factors. Our data are consistent with ROS/KEAP1-mediated NRF2 activation, so we modulated NRF2 activity by genetic overexpression of KEAP1. Indeed, KEAP1 O/E resulted in a significant reduction in expression of NRF2 target genes and, importantly, a 56% inhibition of the HIFα TFAR peak during iPSC reprogramming compared with controls at day 11. In support of our hypothesis, either NRF2 overexpression or ROS activation with deta NONOate resulted in increased HIFα activation during reprogramming. These data therefore place NRF2 upstream of HIFα activation during iPSC reprogramming. Furthermore, KEAP1 O/E-mediated NRF2 inhibition resulted in reduced glycolytic activation, whereas NRF2 activation resulted in increased glycolysis. This demonstrates that NRF2-HIFα co-operation promotes the metabolic switch to glycolytic energy production. Moreover, KEAP1 O/E-mediated NRF2 inhibition reduced iPSC colony formation consistent with the observations of Jang et al. (2014), who showed that NRF2 shRNA transcript knockdown decreased reprogramming efficiency. NRF2 has been shown to activate HIF1α via TRX1 in lung adenocarcinoma A549 cells (Malec et al., 2010). We speculate that the same mechanism may be occurring during iPSC reprogramming, as we show here that TRX1 is significantly increased during this process. Our demonstration of the central role of NRF2 in iPSC reprogramming is consistent with the observation that cells with higher levels of OXPHOS reprogram more readily (Esteban et al., 2010, Liu et al., 2013), possibly due to increased NRF2 activity in these cells.

In summary, we present evidence that the metabolic changes occurring throughout iPSC reprogramming are more complex than a simple switch from one predominant form of energy production to another. Instead, they constitute a series of intermediate steps involving an initial increase in OXPHOS and diversion of glucose from glycolysis to the PPP, before the well-documented “metabolic switch” in which cells increase glycolysis and decrease OXPHOS. We also demonstrate unequivocally that molecules controlling the cellular redox state and metabolic states work together to facilitate the ultimate transition from somatic to pluripotent cellular metabolism. Elucidation of the molecular interactions between NF-κB, AP-1, NRF2, and HIFα transcription factors and their potential roles in the metabolic switch therefore warrants further investigation since manipulation of the redox state using small molecules has potential to improve iPSC reprogramming efficiency. Finally, these data collectively add weight to the emerging concept that, at least in the pre-colony forming initiation stages, iPSC reprogramming is a stage-wise deterministic process with quality-control checkpoints. This process shows intriguing similarities with tumor initiation that warrants further investigation in order to guarantee the development of safe and efficacious regenerative medicine approaches.

## Experimental Procedures

### iPSC Reprogramming of hDFs

hDFs were transduced with a MOI of <10 of the appropriate NLuc lentiviral vector or control pLNT-SFFV-VLuc vector 7 days prior to reprogramming. Transduced hDFs were then dissociated using Trypsin/EDTA (Sigma) and 5 × 10^5^ cells transfected with either 2 μg of each episomal reprogramming plasmid (pCXLE-hUL, pCXLE-hSK, pCXLE-hOCT3/4-shp53-F, and pCXWB-EBNA1; Addgene) or 8 μg of the episomal control plasmid in electroporation buffer (Lonza) using an Amaxa Nucleofector according to the manufacturer’s instructions. After transfection, cells were seeded on a six-well plate in complete DMEM (DMEM supplemented with 10% fetal bovine serum [FBS], 1% non-essential amino acids [NEAAs], 4% 200 mM L-glutamine, and 1% penicillin/streptomycin [P/S]; all Sigma-Aldrich) without P/S, and medium was changed every 2 days. At 8 days after transfection, 3 × 10^4^ cells were seeded per well in triplicate in a six-well plate containing mitotically inactivated fibroblasts (MEFs) in iPSC media (DMEM/F12 containing Glutamax supplemented with 20% knockout serum replacement, 0.1 mM β-mercaptoethanol [all Life Technologies] 1% P/S and 1% NEAA and 10 ng/ml FGF2 [R&D Systems]). Medium was replenished every 2 days, and samples were taken for luciferase assays as described. Each experiment was repeated at least twice.

Appropriate hDFs were treated with 50 μM deta NONOate (ENZO life sciences) on days 1–6 or NRF2 O/E adenovirus (a kind gift from Dr. Stephen White) or the adenoviral control (AV CTL) on day 2.

### Luciferase Assays

Supernatant was collected from triplicate wells of cells at the appropriate time points, and 20 μl was transferred to 20 μl assay buffer (25 mM Tris Phosphate [pH 7.8] containing 1% BSA and 30% glycerol; all Sigma-Aldrich) in a white-bottomed 96-well plate (Corning) in technical triplicates. VLuc samples were assayed detecting photonic emissions at 460 nm after addition of 5 nM vargulin (Gold Biotechnology) and NLuc photonic emissions at 454 nm after addition of 1 mM coelenterazine (Gold Biotechnology) using a Promega GloMax 96 luminometer.

### Data Analysis

The following equation was used to analyze the data:

$$\frac{\left(\text{TFAR}\;\text{Nluc}\;\text{iPSC/SFFV}\;\text{Vluc}\;\text{iPSC}\right)}{\left(\text{TFAR}\;\text{Nluc}\;\text{control/SFFV}\;\text{Vluc}\;\text{control}\right)}.$$

These values were then plotted graphically against the number of days post-transfection.

### ATP Production Assays

Cells were plated on tissue culture-treated white-bottomed 96-well plates (Corning) in triplicate per treatment. Cells were washed with complete DMEM before appropriate compounds were added; 3 μM IAA (Sigma) was used to block glycolysis, and 5 μM oligomycin A (Sigma) was used to block OXPHOS. The plate was incubated at 37°C for 1 hr before being analyzed using a Cell Titer Glo Luminescent Assay (Promega) according to the manufacturer’s instructions. The inverse of the mean value for levels of ATP produced in the presence of the drugs divided by the total ATP produced was then plotted graphically.

### ROS Determination

Cells were washed with PBS and incubated in 5 μM DCF-DA in complete DMEM for 30 min before being washed with PBS and dissociated with Trypsin/EDTA. Cells were then resuspended in PBS and the levels of fluorescence in the FL1 channel analyzed using a FACSCalibur (BD Biosciences). The excitation and emission wavelengths were set to 490 and 535 nm, respectively.

## Author Contributions

T.R.M. and K.E.H. formulated the broad concept. K.E.H. and J.M.K.M.D. designed and generated vectors. K.E.H. and S.J. performed all iPSC experiments, and K.E.H. performed all molecular biological experiments. K.E.H., V.N.K., and M.R.D. performed metabolic assays. E.F. and J.P.B. performed the PPP and glycolytic flux experiments. S.N.W. performed in vivo teratoma experiments. J.R.W. generated the ICAM1 TFAR construct. L.M.F., K.E.H., and P.J.K. analyzed the RNA-seq data, and K.E.H. and T.R.M. prepared the manuscript.

## Supplemental Information

Document S1. Supplemental Experimental Procedures, Figures S1–S4, and Tables S1–S4

Document S2. Article plus Supplemental Information

## References

[bib1] Baird L., Llères D., Swift S., Dinkova-Kostova A.T. (2013). Regulatory flexibility in the Nrf2-mediated stress response is conferred by conformational cycling of the Keap1-Nrf2 protein complex. Proc. Natl. Acad. Sci. USA.

[bib2] Buckley S.M.K., Delhove J.M.K.M., Perocheau D.P., Karda R., Rahim A.A., Howe S.J., Ward N.J., Birrell M.A., Belvisi M.G., Arbuthnot P. (2015). In vivo bioimaging with tissue-specific transcription factor activated luciferase reporters. Sci. Rep..

[bib3] Chen H.-F., Kuo H.-C., Lin S.-P., Chien C.-L., Chiang M.-S., Ho H.-N. (2010). Hypoxic culture maintains self-renewal and enhances embryoid body formation of human embryonic stem cells. Tissue Eng. Part A.

[bib4] Cho Y.M., Kwon S., Pak Y.K., Seol H.W., Choi Y.M., Park J., Park K.S., Lee H.K. (2006). Dynamic changes in mitochondrial biogenesis and antioxidant enzymes during the spontaneous differentiation of human embryonic stem cells. Biochem. Biophys. Res. Commun..

[bib5] Chowdhry S., Zhang Y., McMahon M., Sutherland C., Cuadrado A., Hayes J.D. (2013). Nrf2 is controlled by two distinct β-TrCP recognition motifs in its Neh6 domain, one of which can be modulated by GSK-3 activity. Oncogene.

[bib6] Danet G.H., Pan Y., Luongo J.L., Bonnet D.A., Simon M.C. (2003). Expansion of human SCID-repopulating cells under hypoxic conditions. J. Clin. Invest..

[bib7] Esteban M.A., Wang T., Qin B., Yang J., Qin D., Cai J., Li W., Weng Z., Chen J., Ni S. (2010). Vitamin C enhances the generation of mouse and human induced pluripotent stem cells. Cell Stem Cell.

[bib8] Ezashi T., Das P., Roberts R.M. (2005). Low O2 tensions and the prevention of differentiation of hES cells. Proc. Natl. Acad. Sci. USA.

[bib9] Finley L.W.S., Carracedo A., Lee J., Souza A., Egia A., Zhang J., Teruya-Feldstein J., Moreira P.I., Cardoso S.M., Clish C.B. (2011). SIRT3 opposes reprogramming of cancer cell metabolism through HIF1α destabilization. Cancer Cell.

[bib10] Folmes C.D.L., Nelson T.J., Martinez-Fernandez A., Arrell D.K., Lindor J.Z., Dzeja P.P., Ikeda Y., Perez-Terzic C., Terzic A. (2011). Somatic oxidative bioenergetics transitions into pluripotency-dependent glycolysis to facilitate nuclear reprogramming. Cell Metab..

[bib11] Golipour A., David L., Liu Y., Jayakumaran G., Hirsch C.L., Trcka D., Wrana J.L. (2012). A late transition in somatic cell reprogramming requires regulators distinct from the pluripotency network. Cell Stem Cell.

[bib12] Gonchar O., Mankovska I. (2010). Antioxidant System in Adaptation to Intermittent Hypoxia. J. Biol. Sci..

[bib13] Hanna J., Saha K., Pando B., van Zon J., Lengner C.J., Creyghton M.P., van Oudenaarden A., Jaenisch R. (2009). Direct cell reprogramming is a stochastic process amenable to acceleration. Nature.

[bib14] Hansson J., Rafiee M.R., Reiland S., Polo J.M., Gehring J., Okawa S., Huber W., Hochedlinger K., Krijgsveld J. (2012). Highly coordinated proteome dynamics during reprogramming of somatic cells to pluripotency. Cell Rep..

[bib15] Hayashi K., Dan K., Goto F., Tshuchihashi N., Nomura Y., Fujioka M., Kanzaki S., Ogawa K. (2015). The autophagy pathway maintained signaling crosstalk with the Keap1-Nrf2 system through p62 in auditory cells under oxidative stress. Cell. Signal..

[bib16] Herrero-Mendez A., Almeida A., Fernández E., Maestre C., Moncada S., Bolaños J.P. (2009). The bioenergetic and antioxidant status of neurons is controlled by continuous degradation of a key glycolytic enzyme by APC/C-Cdh1. Nat. Cell Biol..

[bib17] Ichida J.K., Tcw J., Williams L.A., Carter A.C., Shi Y., Moura M.T., Ziller M., Singh S., Amabile G., Bock C. (2014). Notch inhibition allows oncogene-independent generation of iPS cells. Nat. Chem. Biol..

[bib18] Ichimura Y., Waguri S., Sou Y.-S., Kageyama S., Hasegawa J., Ishimura R., Saito T., Yang Y., Kouno T., Fukutomi T. (2013). Phosphorylation of p62 activates the Keap1-Nrf2 pathway during selective autophagy. Mol. Cell.

[bib19] Jacobson J., Duchen M.R., Hothersall J., Clark J.B., Heales S.J.R. (2005). Induction of mitochondrial oxidative stress in astrocytes by nitric oxide precedes disruption of energy metabolism. J. Neurochem..

[bib20] Jang J., Wang Y., Kim H.-S., Lalli M.A., Kosik K.S. (2014). Nrf2, a regulator of the proteasome, controls self-renewal and pluripotency in human embryonic stem cells. Stem Cells.

[bib21] Ji J., Sharma V., Qi S., Guarch M.E., Zhao P., Luo Z., Fan W., Wang Y., Mbabaali F., Neculai D. (2014). Antioxidant supplementation reduces genomic aberrations in human induced pluripotent stem cells. Stem Cell Reports.

[bib22] Larrabee M.G. (1990). Evaluation of the pentose phosphate pathway from 14CO2 data. Fallibility of a classic equation when applied to non-homogeneous tissues. Biochem. J..

[bib23] Liu W., Long Q., Chen K., Li S., Xiang G., Chen S., Liu X., Li Y., Yang L., Dong D. (2013). Mitochondrial metabolism transition cooperates with nuclear reprogramming during induced pluripotent stem cell generation. Biochem. Biophys. Res. Commun..

[bib24] Lonergan T., Brenner C., Bavister B. (2006). Differentiation-related changes in mitochondrial properties as indicators of stem cell competence. J. Cell. Physiol..

[bib25] Mah N., Wang Y., Liao M.-C., Prigione A., Jozefczuk J., Lichtner B., Wolfrum K., Haltmeier M., Flöttmann M., Schaefer M. (2011). Molecular insights into reprogramming-initiation events mediated by the OSKM gene regulatory network. PLoS ONE.

[bib26] Malec V., Gottschald O.R., Li S., Rose F., Seeger W., Hänze J. (2010). HIF-1 alpha signaling is augmented during intermittent hypoxia by induction of the Nrf2 pathway in NOX1-expressing adenocarcinoma A549 cells. Free Radic. Biol. Med..

[bib27] Manganelli G., Fico A., Masullo U., Pizzolongo F., Cimmino A., Filosa S. (2012). Modulation of the pentose phosphate pathway induces endodermal differentiation in embryonic stem cells. PLoS ONE.

[bib28] Mathieu J., Zhou W., Xing Y., Sperber H., Ferreccio A., Agoston Z., Kuppusamy K.T., Moon R.T., Ruohola-Baker H. (2014). Hypoxia-inducible factors have distinct and stage-specific roles during reprogramming of human cells to pluripotency. Cell Stem Cell.

[bib29] McMahon M., Thomas N., Itoh K., Yamamoto M., Hayes J.D. (2006). Dimerization of substrate adaptors can facilitate cullin-mediated ubiquitylation of proteins by a “tethering” mechanism: a two-site interaction model for the Nrf2-Keap1 complex. J. Biol. Chem..

[bib30] Mitsuishi Y., Taguchi K., Kawatani Y., Shibata T., Nukiwa T., Aburatani H., Yamamoto M., Motohashi H. (2012). Nrf2 redirects glucose and glutamine into anabolic pathways in metabolic reprogramming. Cancer Cell.

[bib31] Morrison S.J., Csete M., Groves A.K., Melega W., Wold B., Anderson D.J. (2000). Culture in reduced levels of oxygen promotes clonogenic sympathoadrenal differentiation by isolated neural crest stem cells. J. Neurosci..

[bib32] O’Malley J., Skylaki S., Iwabuchi K.A., Chantzoura E., Ruetz T., Johnsson A., Tomlinson S.R., Linnarsson S., Kaji K. (2013). High-resolution analysis with novel cell-surface markers identifies routes to iPS cells. Nature.

[bib33] Palomäki S., Pietilä M., Laitinen S., Pesälä J., Sormunen R., Lehenkari P., Koivunen P. (2013). HIF-1α is upregulated in human mesenchymal stem cells. Stem Cells.

[bib34] Panopoulos A.D., Yanes O., Ruiz S., Kida Y.S., Diep D., Tautenhahn R., Herrerías A., Batchelder E.M., Plongthongkum N., Lutz M. (2012). The metabolome of induced pluripotent stem cells reveals metabolic changes occurring in somatic cell reprogramming. Cell Res..

[bib35] Park K.-K., Jung E., Chon S.-K., Seo M., Kim H.W., Park T. (2003). Finding of TRE (TPA responsive element) in the sequence of human taurine transporter promoter. Adv. Exp. Med. Biol..

[bib36] Park S.-J., Yeo H.C., Kang N.-Y., Kim H., Lin J., Ha H.-H., Vendrell M., Lee J.-S., Chandran Y., Lee D.-Y. (2014). Mechanistic elements and critical factors of cellular reprogramming revealed by stepwise global gene expression analyses. Stem Cell Res. (Amst.).

[bib37] Polo J.M., Anderssen E., Walsh R.M., Schwarz B.A., Nefzger C.M., Lim S.M., Borkent M., Apostolou E., Alaei S., Cloutier J. (2012). A molecular roadmap of reprogramming somatic cells into iPS cells. Cell.

[bib38] Prigione A., Fauler B., Lurz R., Lehrach H., Adjaye J. (2010). The senescence-related mitochondrial/oxidative stress pathway is repressed in human induced pluripotent stem cells. Stem Cells.

[bib39] Prigione A., Rohwer N., Hoffmann S., Mlody B., Drews K., Bukowiecki R., Blümlein K., Wanker E.E., Ralser M., Cramer T., Adjaye J. (2014). HIF1α modulates cell fate reprogramming through early glycolytic shift and upregulation of PDK1-3 and PKM2. Stem Cells.

[bib40] Reuter S., Gupta S.C., Chaturvedi M.M., Aggarwal B.B. (2010). Oxidative stress, inflammation, and cancer: how are they linked?. Free Radic. Biol. Med..

[bib41] Samavarchi-Tehrani P., Golipour A., David L., Sung H.K., Beyer T.A., Datti A., Woltjen K., Nagy A., Wrana J.L. (2010). Functional genomics reveals a BMP-driven mesenchymal-to-epithelial transition in the initiation of somatic cell reprogramming. Cell Stem Cell.

[bib42] Shimada H., Hashimoto Y., Nakada A., Shigeno K., Nakamura T. (2012). Accelerated generation of human induced pluripotent stem cells with retroviral transduction and chemical inhibitors under physiological hypoxia. Biochem. Biophys. Res. Commun..

[bib43] Singh A., Happel C., Manna S.K., Acquaah-Mensah G., Carrerero J., Kumar S., Nasipuri P., Krausz K.W., Wakabayashi N., Dewi R. (2013). Transcription factor NRF2 regulates miR-1 and miR-206 to drive tumorigenesis. J. Clin. Invest..

[bib44] Studer L., Csete M., Lee S.H., Kabbani N., Walikonis J., Wold B., McKay R. (2000). Enhanced proliferation, survival, and dopaminergic differentiation of CNS precursors in lowered oxygen. J. Neurosci..

[bib45] Takahashi K., Yamanaka S. (2006). Induction of pluripotent stem cells from mouse embryonic and adult fibroblast cultures by defined factors. Cell.

[bib46] Tsai J.J., Dudakov J.A., Takahashi K., Shieh J.-H., Velardi E., Holland A.M., Singer N.V., West M.L., Smith O.M., Young L.F. (2013). Nrf2 regulates haematopoietic stem cell function. Nat. Cell Biol..

[bib47] Varum S., Rodrigues A.S., Moura M.B., Momcilovic O., Easley C.A., Ramalho-Santos J., Van Houten B., Schatten G. (2011). Energy metabolism in human pluripotent stem cells and their differentiated counterparts. PLoS ONE.

[bib48] Yoshida Y., Takahashi K., Okita K., Ichisaka T., Yamanaka S. (2009). Hypoxia enhances the generation of induced pluripotent stem cells. Cell Stem Cell.

[bib49] Yu J., Hu K., Smuga-Otto K., Tian S., Stewart R., Slukvin I.I., Thomson J.A. (2009). Human induced pluripotent stem cells free of vector and transgene sequences. Science.

[bib50] Zhang J., Khvorostov I., Hong J.S., Oktay Y., Vergnes L., Nuebel E., Wahjudi P.N., Setoguchi K., Wang G., Do A. (2011). UCP2 regulates energy metabolism and differentiation potential of human pluripotent stem cells. EMBO J..

[bib51] Zhang X., Yalcin S., Lee D.-F., Yeh T.-Y.J., Lee S.-M., Su J., Mungamuri S.K., Rimmelé P., Kennedy M., Sellers R. (2011). FOXO1 is an essential regulator of pluripotency in human embryonic stem cells. Nat. Cell Biol..

[bib52] Zhu L., Zhang S., Jin Y. (2014). Foxd3 suppresses NFAT-mediated differentiation to maintain self-renewal of embryonic stem cells. EMBO Rep..

