# Supplementary material for: NRF2 Orchestrates the Metabolic Shift during Induced Pluripotent Stem Cell Reprogramming
Source: Cell Rep. 2016 Feb 18;14(8):1883–91. doi: 10.1016/j.celrep.2016.02.003 (PMC4785773; doi:10.1016/j.celrep.2016.02.003)
Supplement: Document S1. Supplemental Experimental Procedures, Figures S1–S4, and Tables S1–S4 [file mmc1.pdf]

Cell Reports, Volume *14*

Supplemental Information

NRF2 Orchestrates the Metabolic Shift during Induced Pluripotent Stem Cell Reprogramming

Kate E. Hawkins, Shona Joy, Juliette M.K.M. Delhove, Vassilios N. Kotiadis, Emilio Fernandez, Lorna M. Fitzpatrick, James R. Whiteford, Peter J. King, Juan P. Bolanos, Michael R. Duchen, Simon N. Waddington, and Tristan R. McKay

Supplemental Information Inventory

Supplemental Figures

Figure S1. Related to Figure 1.

Figure S2. Related to Figure 1.

Figure S3. Related to Figures 1, 2 and 3.

Figure S4. Related to Figure 4.

Supplemental Tables

Table S1. TFAR Agonists and Antagonists.

Table S2. Cloning PCR Primers.

Table S3. qRT PCR Primers.

Table S4. RT PCR Primers.

Supplemental Experimental Procedures.

Supplemental References.

Supplemental Figures:

Figure S1. Related to Figure 1. (A)(i) Phase contrast images to show pluripotent morphology of iPSCs. (ii) RT-PCR to show pluripotency-associated transcript expression in iPSCs. (B) Immunofluorescent staining to show markers of the 3 germ layers following EB differentiation. (C) H&E staining of teratoma sections to show the 3 germ layers. (D)(i) NLuc lentiviral vector schematic (ii) VLuc lentiviral vector schematic. Scale bars: 100 μm. hESC: human embryonic stem cell, MP: minimal promoter, EB: embryoid body, H&E: Haematoxylin and Eosin.

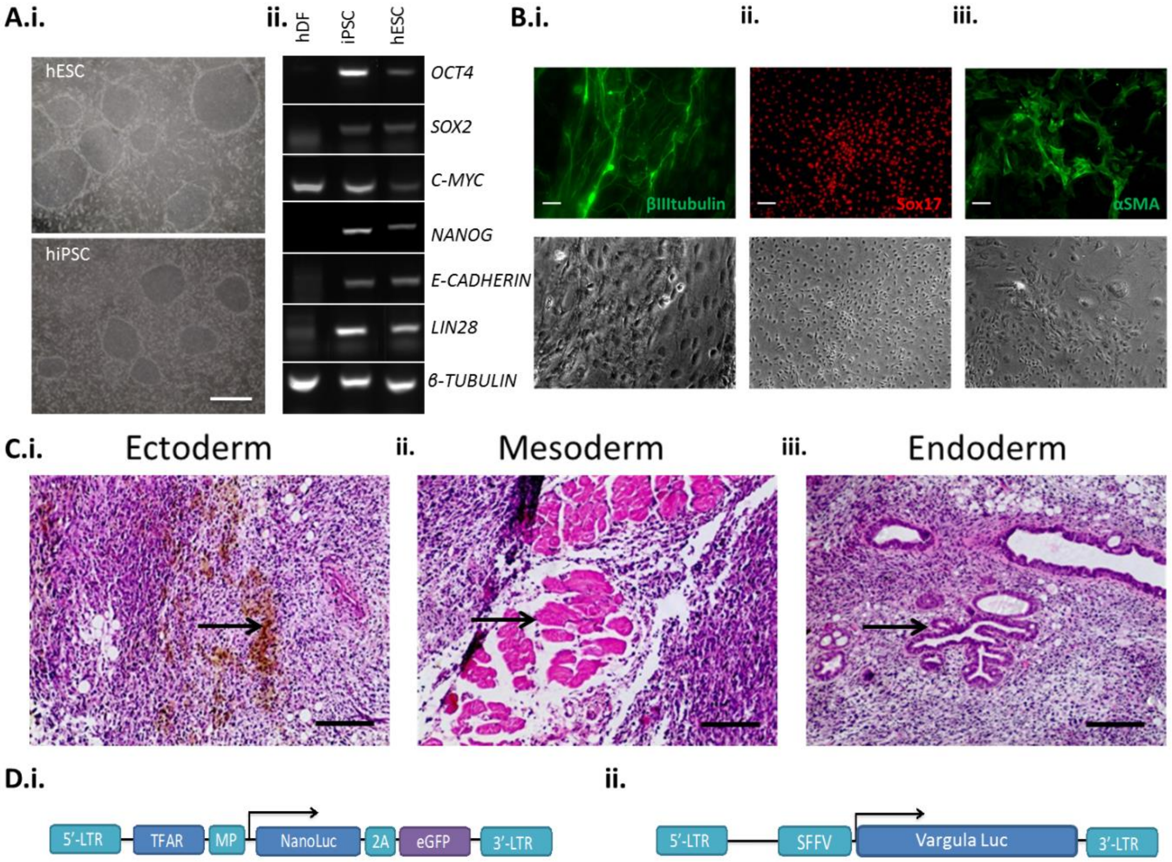

Figure S2. Related to Figure 1. eGFP images and luciferase assays for TFAR activation. Scale bars: 100 µm. Error bars: SEM for 3 biological replicates.

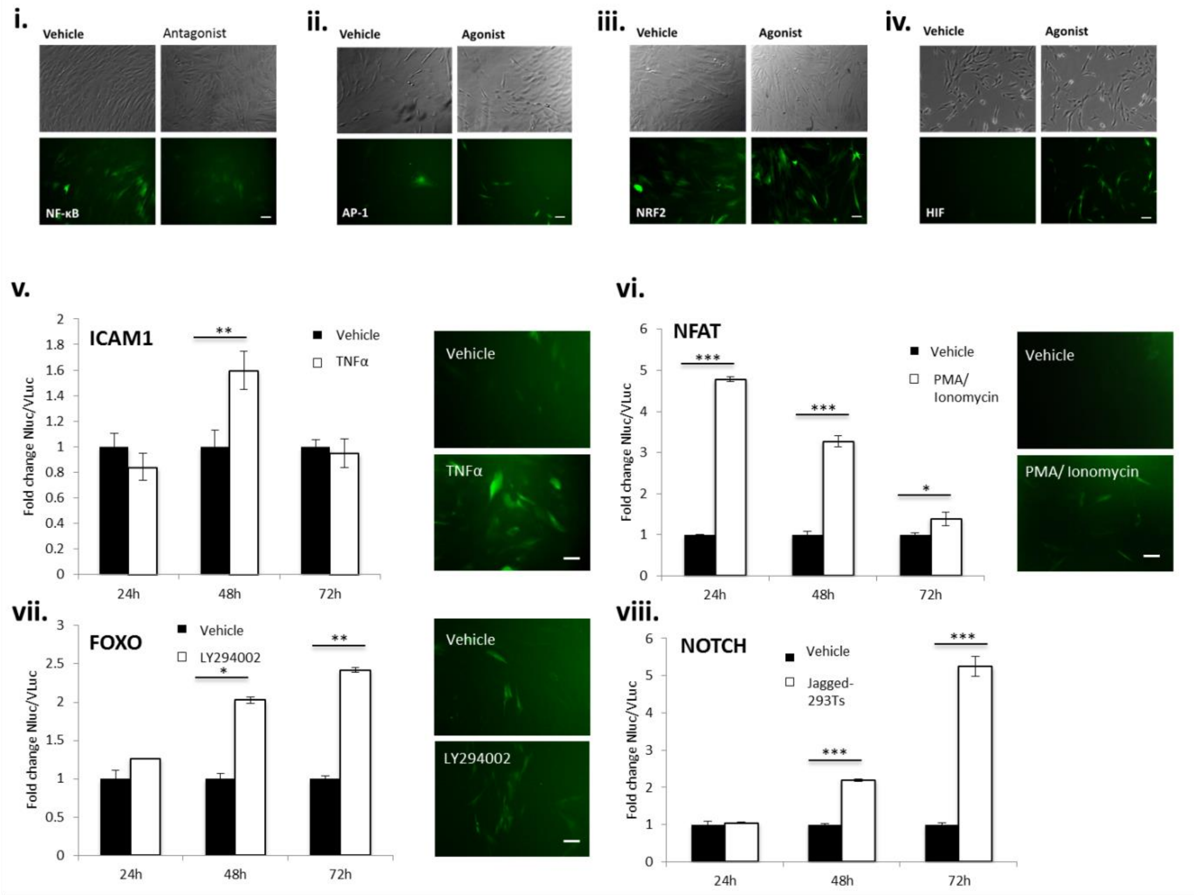

Figure S3. Related to Figures 1, 2 and 3. (i-iv) TFAR luciferase data. (v) qPCR analysis of NF-κB, AP-1, HIFα and NRF2 target gene expression at day 2 of iPSC reprogramming. (vi) Immunofluorescent staining to show that c-Fos is localised in the nucleus of pre-iPSCs but largely excluded from the nucleus of control cells at day 4 of iPSC reprogramming. (vii) qPCR analysis to show levels of KEAP1 transcripts at day 8 of iPSC reprogramming. (viii) Oxygen consumption in pre-iPSCs and CTL cells following injection of the Complex V inhibitor oligomycin A, the uncoupler FCCP and antimycin A. N=1. Scale bars: 100 μm. Error bars: SEM for 3 biological replicates. FCCP: carbonyl cyanide 4-(trifluoromethoxy) phenylhydrazone.

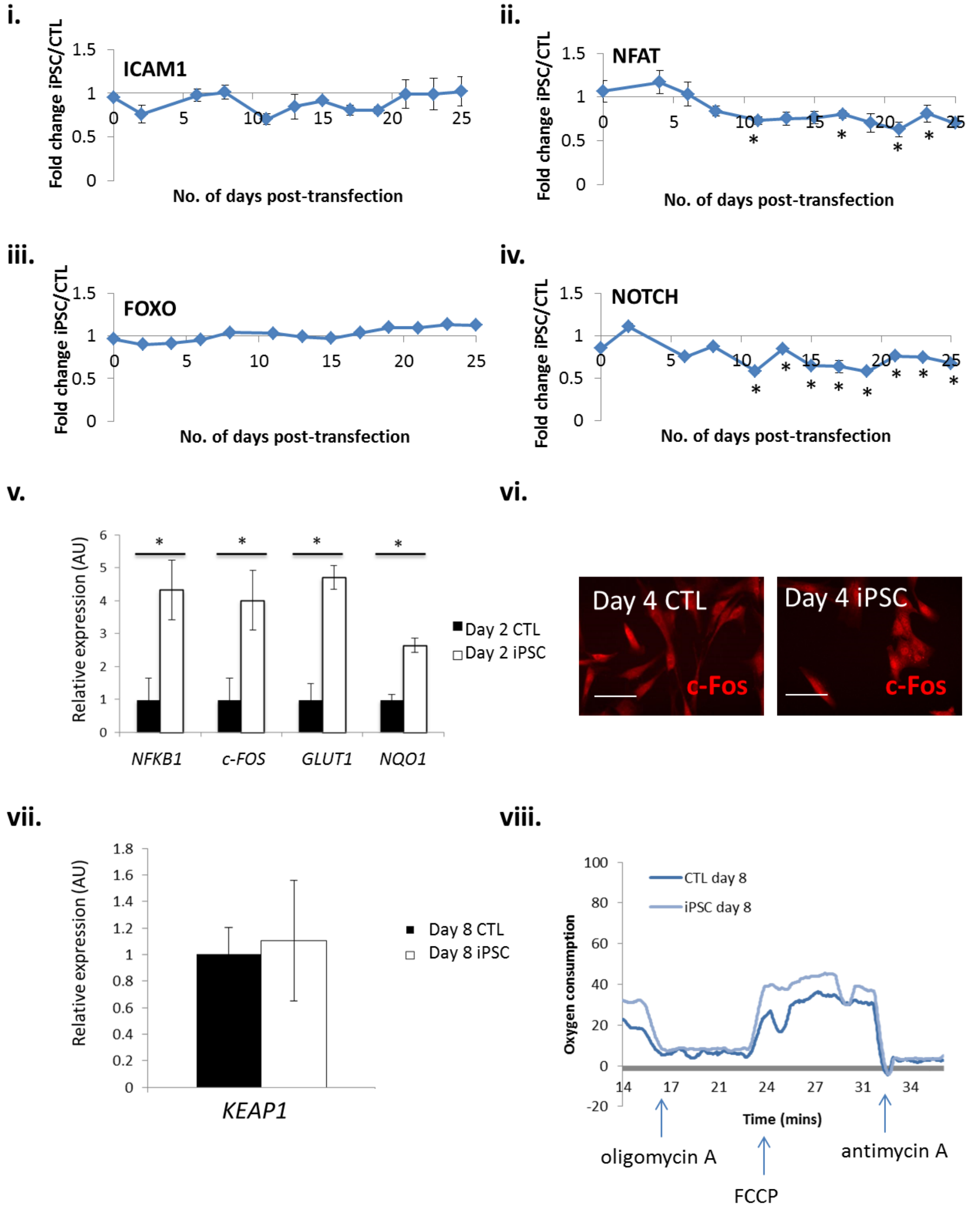

Figure S4. Related to Figure 4. (i) ROS assay to show levels of ROS production at day 4 of reprogramming. (ii-iii) Graphs to show fold change of normalised TFAR activity for iPSCs exposed to 50 µM deta NONOate compared to control cells. (iv) Luciferase assay to show levels of NRF2 activation in response to an NRF2 O/E adenovirus. (v-vi) Graphs to show fold change of normalised TFAR activity for hDFs exposed to an NRF2 O/E adenovirus or its control (AV CTL) throughout reprogramming. (vii) Lactate assay to show increased lactate production in response to 50 µM deta NONOate and (viii) NRF2 O/E compared to controls. Error bars: SEM for 3 biological replicates. ROS: reactive oxygen species, TFAR: transcription factor activated reporter, O/E: overexpression, TPP: triphenylphosphonium. *p<0.05, **p<0.01, ***p<0.005.

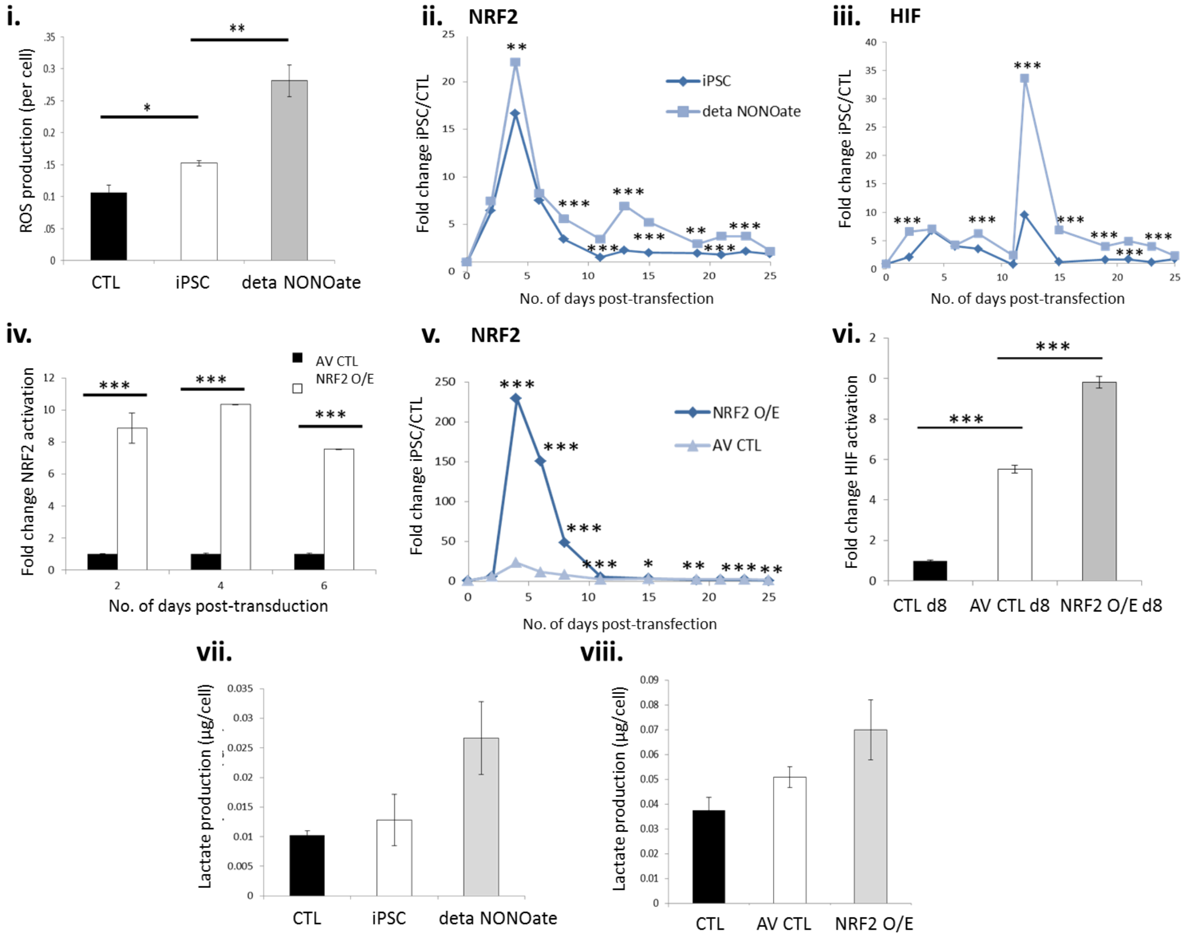

Supplemental Tables

Table S1. TFAR agonists and antagonists.

**TFAR**	**Agonist/Antagonist**	**Supplier**
NRF2	Sulfurophane (10 μM)	Sigma
HIFα	1% oxygen	N/A
AP-1	Phorbol myristate acetate (PMA; 10 ng/ml)	Sigma
NF-κB	Dexamethasone (100 nM)	Sigma
NOTCH	Jagged 1 expressing 293T cells	N/A
NFAT	PMA and Ionomycin (0.5 μM)	Sigma, Tocris
FOXO	LY294002 (20 mM)	Merck Millipore
ICAM1	TNFα (10 ng/ml)	Sigma

Table S2. Cloning PCR primers.

**Primer target**	**Forward sequence**	**Reverse sequence**
NLuc/eGFP	CACACTCGAGGCCACCATGAACTCCTTCTCCACAAGCG	GATGACGCGTTTACTTGTACAGCTCGTCCATGCCG
ICAM1 promoter	TTAATTGAATTCGATTCCCTTGAGGTCACTCT	TATATACTCGAGTGCAGGGCCAGGGCAAAGT

Table S3. qRT PCR primers.

Primer target	Forward sequence (5'-3')	Reverse sequence (5'-3')
NRF2	ATAGCTGAGCCCAGTATC	CATGCACGTGAGTGCTCT
TRX1	CAGATCGAGAGCAAGACTGCTTTTC	CTTATTGGCTCCAGAAAATTCACCC
NQO1	GGGCAAGTCCATCCCAACTG	GCAAGTCAGGGAAGCCTGGA
HIF1α	CCAGTTACGTTCCTTCGATCAGT	TTTGAGGACTTGCGCTTTCA
HIF2α	GTCTCTCCACCCCATGTCTC	GGTTCTTCATCCGTTTCCAC
GLUT1	AACTCTTCAGCCAGGGTCCAC	CACAGTGAAGATGATGAAGAC
NF-κB1	TGCCAACAGATGGCCCATAC	TGTTCTTTTCACTAGAGGCACCA
c-FOS	AGAATCCGAAGGGAAAGGAA	CTTCTCCTTCAGCAGGTTGG
β-ACTIN	CGGGACCTGACTGACTACC	TGAAGGTAGTTTCGTGGATGC
HO-1	CATGACACCAAGGACCAGAG	AGTGTAAGGACCCATCGGAG
GCLC	ATGGAGGTGCAATTAACAGAC	ACTGCATTGCCACCTTTGCA
SRXN1	CCCATCGATGTCCTCTGGATCAAA	AGGTACACCCTTAGGTCTGAGAGA
KEAP1	TGGCTGTATCCACCACAACAG	CATTCGCCACTAATTCCTCTC

Table S4. RT PCR primers.

Transcript	Forward sequence (5'-3')	Reverse sequence (5'-3')
OCT4	GTCTCCTTTCTCAGGGGGAC	CAAAAACCCTGGCACAAACT
SOX2	GACCAGCTCGCAGACCTACAT	TACCTCTTCCTCCCACTCCA
c-MYC	CAAAAACATCATCATCCAGGAC	CTCCACCTCCAGCTTGTACC
NANOG	AGCCTCTACTCTTCCTACCACC	TCCAAAGCAGCCTCCAAGTC
E-CADHERIN	TGCCCAGAAAATGAAAAAGG	GAACGCATTGCCACATAC
LIN28	AAGCGCAGATCAAAAGGAGA	CACTTCTGCCAGAGCATCAG
β-TUBULIN	ACATCCAGGCTGGTCAGTGT	CCCAGGTTCTAGATCCACCA

Supplemental Experimental Procedures

hDF Isolation and Culture

Normal excess skin samples were obtained following informed consent in accordance with Local Research Ethics approval (LREC:14/NS/1073) from patients at St George's University of London. Fatty tissue was removed and skin sections were cut into small pieces before being washed for 1 minute in 0.25% iodine (VWR International), 1 minute in 70% ethanol, 10 minutes in 50 µg/ml gentamycin then submerged, epidermis down in 1% dispase overnight at 4˚C. The epidermis was removed and the dermis minced and placed in collagenase at 37˚C for 2 hours with shaking every 20 minutes. Supernatant was removed and cells pelleted and resuspended in Complete DMEM and plated onto tissue culture-treated 6 well plates. Unless otherwise specified, cells were maintained at 37˚C with ambient oxygen and 5% CO_2. When necessary, cells were passaged using Trypsin/EDTA. hDFs used in reprogramming experiments did not exceed 5 serial passages.

Validation of TFAR Vectors

hDFs were transduced with a MOI of <10 of the appropriate lentiviral vector. Four days later, transduced hDFs were seeded in 24 well plates and agonists/antagonists added as detailed in Table 1. Cells were also transduced with pLNT-SFFV-VLuc to control for cellular proliferation over time. hDFs transduced with the HIFα TFAR were activated by exposure to 1% oxygen in a hypoxic chamber. Conditioned medium containing secreted luciferase was collected at 24, 48 and 72 hours post-seeding and luciferase activity assayed as described in the main text.

Lentiviral Cloning and Production

NanoLuc® luciferase (NLuc) was amplified using pLNL1.3 (Promega) as template and fused to 2A eGFP using overlap extension PCR. This product was then amplified using NLuc(F) and NLuc(R) primers detailed in Table 2, subcloned into pENTR-no ccdB (Addgene) in the reverse orientation using *Eco*RV and cloned into pLNT-Gateway(GW)-MCS (A kind gift from Dr Steven Howe) using *Xho*I. TFAR consensus sequences, *de novo* synthesized and cloned into pENTR (Life Technologies) modified to contain the adenoviral E1A minimal promoter (pENTR-1A) by Aldevron were then shuttled into pLNT-GW-NLuc/eGFP using Gateway® cloning (Life Technologies) with the exception of ICAM1. To generate the ICAM1 reporter construct, the 1500 bp sequence immediately preceding the start codon of murine ICAM1 was amplified by PCR using C57BL6 mouse genomic DNA as a template. Primer sequences are shown in Table 2. The PCR product was digested with *Eco*RI and *Xho*I and ligated into the corresponding sites of pENTR-1A, then shuttled into pLNT-GW-NLuc/eGFP using Gateway® cloning. The control constitutively active lentiviral vector pLNT-SFFV-VLuc was generated by cloning Vargula luciferase (VLuc) from pCMV-VargLuc (Targeting Systems) into pENTR-1A using *Bam*HI and *Not*I restriction sites. VLuc was then shuttled into pLNT-SFFV-GW using Gateway® cloning. Similarly, KEAP1 was cloned from FLAG-KEAP1 (Addgene) into pENTR-1A using *BamHI* and *NotI* restriction sites then shuttled into pLNT-SFFV-GW using Gateway® cloning. The pLNT-SFFV-MCS plasmid containing a multiple cloning site in the place of KEAP1 was used as a control (LNT CTL). The episomal control plasmid was generated by excising the transgenes from pCXLE-hSK using *EcoRI* (New England Biolabs), inactivating the enzyme at 65˚C for 20 minutes and then ligating the backbone using Quick ligase (NEB).

Lentivirus was produced as previously described (Buckley et al., 2015). Briefly, HEK293 cells were grown to 80% confluency and transfected using polyethylenimine precomplexed with 50 µg of the appropriate lentiviral vector along with 17.5 µg VSVg envelope plasmid (pMD.G2) and 32.5 µg packaging plasmid (pCMVΔ8.74) in OptiMEM (Life Technologies) for 3 hours prior to re-feeding. Culture medium was then harvested at 48 and 72 hours, filtered through a 0.45 µm PVDF filter (Elkay) and pelleted by centrifugation at 5000 g overnight at 4˚c. The viral pellet was then resuspended in 50 µl OptiMEM and cryopreserved at -80˚C. The viral titer was determined using a p24 assay (Zeptometrix) according to the manufacturer's instructions.

Immunofluorescent Cell Staining

Cells were washed 5 times with phosphate buffered saline (PBS, Sigma) and fixed *in situ* using fresh 4% paraformaldehyde (Thermo Scientific) for 15 minutes at room temperature before being washed with PBS and permeablized with 0.3% Triton (Sigma) in PBS. Cells were then washed with PBS and incubated in blocking solution (0.1%/0.3% Triton in PBS) for 30 minutes at room temperature. Cells were incubated in the appropriate primary antibody at its optimal dilution in blocking solution overnight at 4˚C. Primary antibodies were as follows: anti-OCT4 (1:200), anti-SOX2 (1:100), anti-NANOG (1:100), anti α-SMA (1:200), anti-βIIItubulin

(1:200), anti-Sox17 (1:200, all R&D systems) and anti-c-Fos (1:40, Abcam). Cells were then washed with PBS and incubated with the appropriate Alexa fluor 488-conjugated mouse), Alexa fluor 568-conjugated rabbit (both 1:500, Life Technologies) or Northern Lights goat secondary antibody (1:400, R&D systems) for 1 hour at room temperature in the dark. Cells were then washed and resuspended in PBS containing DAPI (Thermo Scientific) for visualisation on a Nikon inverted fluorescence microscope.

EB Generation

Human iPSCs maintained on MEFs were trypsinized, resuspended in iPSC media and seeded in 6 well ultralow attachment plates (Corning) with 10 µM Y-27632 (Sigma). Medium was changed on day 4 to DMEM + 20% FBS and on day 7 the EBs were plated on tissue culture treated plates coated with 0.1% porcine gelatin (Sigma). EB outgrowths were fixed with 4% PFA 8 days later as described for immunofluorescent cell staining.

qRT-PCR

Total RNA was isolated from cells using the RNeasy kit (QIAgen) according to the manufacturer's instructions. RNA was reverse transcribed to cDNA using random hexamer primers and MLU reverse transcriptase (both Promega) as per the manufacturer's instructions. Triplicate samples containing appropriate primers (0.4 µM, Table 3) were analysed by qPCR as previously described (Soncin et al., 2011) using β-actin as a housekeeping gene. All results show the SEM of 3 independent replicates.

RT-PCR

Total RNA was isolated and reverse transcribed to cDNA as described above. RT-PCR was then performed using 1 µl of the cDNA and amplified for 30 cycles at the optimal primer annealing temperature (all 54˚C, Table 4). Samples were then separated using a 1.5% agarose gel and visualised using a UV Transilluminator.

Teratoma Formation

iPSC colonies were manually passaged before being dissociated using TrypLE (Life Technologies), counted and resuspended at $1x10^6$ cells/ml Matrigel (Corning). 100 µl cell suspension was then injected subcutaneously into a SCID mouse. 6 weeks later, mice were sacrificed and teratomas excised, fixed in 10% formalin overnight, then taken through an ethanol series to 100% and cleared overnight in Histoclear II (National Diagnostics). They were then embedded in paraffin and sectioned at 6µM, transferred onto slides, cleared in Histoclear II, rehydrated and subsequently stained with Haematoxylin (Sigma) and Eosin (VWR; H&E). Finally, samples were dehydrated, cleared in Histoclear II and mounted using DPX media (VWR).

Western Blotting

Cells were scraped into RIPA buffer (Sigma) containing protease inhibitor cocktail tablets (Roche Applied Science) at a concentration of $2x10^7$ cells/ml. The lysate from $4x10^5$ cells was then incubated on ice for 30 minutes before being boiled in reducing conditions. Proteins were separated using SDS-PAGE and electrotransferred onto Hybond-enhanced chemiluminescence (ECL) nitrocellulose membrane (Amersham Biosciences). The membrane was incubated in blocking buffer (5% dry milk in PBS + 0.05% Tween [Sigma, PBST]). The membrane was then incubated in primary antibody (p62 [BD Biosciences] or ATG5 [Cell Signalling]) in blocking buffer at 4˚C overnight. The membrane was washed three times in PBST and incubated with the appropriate HRP-conjugated secondary antibody (Dako, 1:2000) in blocking buffer for 1 hour at room temperature. The membrane was then washed as described previously and developed using ECL (Amersham Biosciences).

Staining for Alkaline Phosphatase activity

Cells were fixed as described above. They were then washed with PBS and incubated with 1 ml/well NBT/BCIP substrate (Sigma) for 30 minutes in the dark before being washed and imaged.

RNA-Seq analysis

RNA was isolated as described above and prepared for Illumina® RNA-seq using the NEBNext® Ultra™ directional RNA library prep kit for Illumina® according to the manufacturer's instructions (NEB). Data can be found here: https://accounts.illumina.com/ by using the username p.j.king@qmul.ac.uk and the password Illuminat1! The list of mitochondrial genes was sourced from the Mitocarta inventory of mammalian mitochondrial genes

(http://www.broadinstitute.org/files/shared/metabolism/mitocarta/human.mitocarta2.0.html) and NRF2 targets were from ChIP seq data (http://amp.pharm.mssm.edu/chea/chipchip.php?info=tf).

Determination of PPP and Glycolytic Flux

PPP flux was measured in 8 cm^2 flasks of adherent cells at 60-70% confluence containing a central microcentrifuge tube with either 0.8 ml benzethonium hydroxide (Sigma) for $^{14}CO_2$ equilibration or 1 ml H_2O for ^3H_2O equilibration. Incubations were carried out in Krebs Ringer Phosphate buffer (145 mM NaCl, 5.7 mM Na_2HPO_4, 4.86 mM KCl, 0.54 mM $CaCl_2$, 1.22 mM $MgSO_4$ pH 7.4) containing 5 mM D-glucose (KPRG buffer) at 37°C. In order to ensure adequate oxygen supply for oxidative metabolism throughout the incubation period, flasks were filled with oxygen before being sealed.

To measure the carbon flux from glucose through the PPP, cells were incubated in KPRG buffer supplemented with 0.5 µCi D-(1-^{14}C]-glucose or [6-^{14}C]-glucose for 90 minutes as previously described (Herrero-Mendez et al., 2009; Rodriguez-Rodriguez et al., 2013). Cellular respiration was then terminated by the addition of 0.2 ml 20% perchloric acid (Merck Millipore) for 40 minutes before the benzethonium hydroxide (containing $^{14}CO_2$) was removed and the radioactivity measured by liquid scintillation counting. PPP flux was calculated as the difference between $^{14}CO_2$ production from [1-^{14}C]-glucose (which decarboxylates through the 6-phosphogluconate dehydrogenase-catalysed reaction) and that of [6-^{14}C]-glucose (which decarboxylates through the tricarboxylic acid cycle) (Herrero-Mendez et al., 2009; Larrabee, 1990). Glycolytic flux was measured by assaying the rate of ^3H_2O production from [3-^{3}H]-glucose using a similar method but instead incubating cells with 5 µCi Glucose-D-[3-^{3}H] in KPRG buffer per flask for 120 minutes as previously described (Herrero-Mendez et al., 2009; Rodriguez-Rodriguez et al., 2013). Respiration was then terminated with perchloric acid and incubating the cells for 96 hours with a microcentrifuge tube containing H_2O suspended above the cells to allow ^3H_2O equilibration. The ^3H_2O was then removed and measured by liquid scintillation counting. Under these experimental conditions, 75% of the produced $^{14}CO_2$ or 28% of the produced ^3H_2O were recovered, as previously established (Rodriguez-Rodriguez et al., 2013).

Oxygen Consumption Measurements

Mitochondrial Oxygen Consumption was analysed using an Oroboros Oxygraph-2k (O2k). Cells were detached using trypsin (Gibco), harvested and resuspended in DMEM medium made from powder (Sigma) with the addition of Sodium Pyruvate (Sigma) 110 mg/L, D-Glucose (Sigma) 1 g/L, 100x Glutamax (Gibco) 10 mL/L, and 20 mM HEPES. Experimental temperature was maintained at 37°C, and oxygen concentration readings from the medium were calibrated according to manufacturer's protocol. After reading basal oxygen consumption measurements (routine Respiration) the addition of oligomycin A 2.5 µM (Sigma) allowed measurements of leak respiration. Titrations of 0.5 µM of with the uncoupler FCCP (Sigma) were used to induce and record maximal uncoupled respiration, and finally addition of 2.5 µM antimycin A (Sigma) allowed the measurement of any non-mitochondrial residual oxygen consumption.

ROS Assays

$1x10^4$ cells were seeded per well of a white-bottomed 96 well plate in triplicate on day 2 of reprogramming. Two days later, the cells were analysed using the Total ROS/Superoxide kit (ENZO life sciences) according to the manufacturer's instructions.

Supplemental References

Soncin, F., Mohamet, L., Ritson, S., Hawkins, K., Bobola, N., Zeef, L., Merry, C.L.R., and Ward, C.M. (2011). E-Cadherin Acts as a Regulator of Transcripts Associated with a Wide Range of Cellular Processes in Mouse Embryonic Stem Cells. PLoS ONE *6*, e21463.

Rodriguez-Rodriguez, P., Fernandez, E., and Bolaños, J.P. (2013). Underestimation of the pentose-phosphate pathway in intact primary neurons as revealed by metabolic flux analysis. J. Cereb. Blood Flow Metab. Off. J. Int. Soc. Cereb. Blood Flow Metab. *33*, 1843–1845.
